# Aspirin eugenol ester affects ileal barrier function, inflammatory response and microbiota in broilers under lipopolysaccharide-induced immune stress conditions

**DOI:** 10.3389/fvets.2024.1401909

**Published:** 2024-05-30

**Authors:** Ruilin Zhang, Dongying Bai, Wenrui Zhen, Xiaodi Hu, Haojie Zhang, Jiale Zhong, Yi Zhang, Koichi Ito, Bingkun Zhang, Yajun Yang, Jianyong Li, Yanbo Ma

**Affiliations:** ^1^Department of Animal Physiology, College of Animal Science and Technology, Henan University of Science and Technology, Luoyang, China; ^2^Henan International Joint Laboratory of Animal Welfare and Health Breeding, College of Animal Science and Technology, Henan University of Science and Technology, Luoyang, China; ^3^Department of Food and Physiological Models, Graduate School of Agricultural and Life Sciences, The University of Tokyo, Ibaraki, Japan; ^4^State Key Laboratory of Animal Nutrition, Department of Animal Nutrition and Feed Science, College of Animal Science and Technology, China Agricultural University, Beijing, China; ^5^Key Lab of New Animal Drug of Gansu Province, Key Lab of Veterinary Pharmaceutical Development of Ministry of Agriculture and Rural Affairs, Lanzhou Institute of Husbandry and Pharmaceutical Science of Chinese Academy of Agricultural Sciences, Lanzhou, China; ^6^Longmen Laboratory, Science and Technology Innovation Center for Completed Set Equipment, Luoyang, China

**Keywords:** immune stress, aspirin eugenol ester, intestinal barrier function, ileal microbiota, broiler

## Abstract

**Aims:**

The aim of this study was to investigate the effects of aspirin eugenol ester (AEE) on ileal immune function in broilers under lipopolysaccharide (LPS)-induced immune stress.

**Methods:**

Two hundred and forty one-day-old male Arbor Acres chicks were randomly divided into four groups (saline, LPS, saline + AEE and LPS + AEE) with six replicates of ten broilers each. The saline group and LPS group were fed the normal diet, while the other two groups received normal diet plus 0.1 g/kg AEE. Broilers in the LPS and LPS + AEE groups were injected intraperitoneally with 0.5 mg/kg B.W LPS in saline for seven consecutive days beginning at 14 days of age, while broilers in the saline and saline + AEE groups were injected with saline only.

**Results:**

The results showed that AEE improved the ileal morphology and increased the ratio of villus height to crypt depth of immune-stressed broilers. LPS-induced immune stress significantly reduced the expression of the genes for the tight junction proteins *occludin*, zonula occludens-1 (*ZO-1*), *claudin-1* and *claudin-2*, in the ileum, while AEE significantly up-regulated the expression of these genes. Compared with the saline group, the LPS-treated chickens showed significantly increased mRNA expression of the inflammatory factors tumor necrosis factor-α (*TNF-α*), interleukin-1β (*IL-1β*), interleukin-6 (*IL-6*), interleukin-10 (*IL-10*), cyclooxygenase-2 (*COX-2*), and microsomal Prostaglandin E Synthesase-1 (*mPGES-1*) in the ileum, while they were significantly decreased by AEE supplementation. In addition, analysis of the ileal bacterial composition showed that compared with saline and LPS + AEE groups, the proportion of Firmicutes and *Lactobacillus* in the LPS group was lower, while the proportion of Proteobacteria and *Escherichia-Shigella* was higher. Similarly, Line Discriminant Analysis Effect Size (LEfSe) analysis showed that compared with the LPS group, *Brevibacillus* was dominant in the saline group, while the LPS + AEE group was rich in *Rhizobium*, *Lachnoclostridium*, Ruminococcaceae, *Faecalibacterium*, *Negativibacillus*, Oscillospiraceae, and *Flavonifractor*.

**Conclusion:**

These results indicate that dietary supplementation with 0.1 g/kg AEE could protect the intestinal health by improving the intestinal villus morphology, enhancing the expression of tight junction genes and alleviating inflammation to resist the immune stress caused by LPS stimulation in broilers, and the mechanism may involve *COX-2*-related signal transduction and improved intestinal microbiota composition.

## 1 Introduction

Under the current intensive broiler breeding program, excessive immune responses, pathogen infection and drug overuse can induce immune stress in chickens (1), resulting in slow growth, decreased immunity and disease resistance (2), and huge economic losses to broiler producers (3). As the organ with the largest area of direct contact with the external environment in the body (4), the intestine not only has the physiological function of food digestion and nutrient absorption in the body (5), but also is an important immune organ (6) for resisting pathogen invasion (7). The ileum is the site where the small intestine and large intestine connect (8), and it plays an important role in the intestinal immune system (9). The occurrence of immune stress in broiler chickens can inhibit the intestinal immune function (10), lead to intestinal inflammation (11), barrier damage (12), and microbial imbalance (13), which negatively affects the birdsʼ health and productivity. Thus, there is an urgent need to discover new safe and efficient anti-stress treatments for poultry production.

Aspirin eugenol ester (AEE) is a new class of non-steroidal anti-inflammatory drugs formed by the combination of aspirin and eugenol through the acyl chloride reaction (14). Compared with the precursor drugs, AEE not only has low toxicity (15), long action time (16), and wide safety range (17), but also reduces the irritation of aspirin on the gastrointestinal tract (18) and enhances the stability of eugenol (19). Studies have shown that AEE has anti-inflammatory (20) and antioxidant (17, 21, 22) effects that are better than either aspirin or eugenol alone (23). In view of the fact that drugs entering the intestine always affect the intestinal microbiota (24), some researchers have conducted experiments with AEE and found that it can indeed alter the composition of the gut microbiota in mice and rats (25, 26) and regulate the metabolomics of cecal contents and feces in rats (27). Moreover, AEE can inhibit the epoxide pathway and reduce the level of the inflammatory mediator cyclooxygenase-2 (*COX-2*) and Prostaglandin E2 (*PGE*_2_) (28). The *COX-2*/*PGE*_2_ signaling pathway can regulate the expression of intestinal tight junction proteins (29), thereby affecting intestinal epithelial barrier function (30–32).

However, the impacts of AEE on growth performance, intestinal mucosal immunity, barrier functions and gut microbiota remain elusive, especially in broiler chickens under immune stress. Lipopolysaccharide (LPS) is one of the most commonly used immune activators (33–35), and intraperitoneal injection can induce immune stress (36, 37). Therefore, this study used intraperitoneal injection of LPS to establish an immune stress model in broiler chickens to explore the protective effect of AEE on intestinal health. Our goal was to obtain theoretical support for the application of AEE in the field of animal husbandry and provide new data on the use of anti-stress drugs in broiler production.

## 2 Materials and methods

### 2.1 Animals and treatments

A total of 240 one-day-old healthy male Arbor Acres broiler chicks were randomly assigned into four groups with six replicates of 10 chicks each. The four groups comprised a saline group, an LPS group, a saline + AEE group and an LPS + AEE group. Broilers are reared in 4-layer stereoscopic superimposed cage, with 6 cages per layer and 10 chickens per cage. Light, temperature and humidity are artificially controlled, and they can drink and eat freely. Broilers in the saline and LPS groups were fed a basal diet, while broilers in the saline + AEE and LPS + AEE groups were fed the basal diet containing 0.1 g/kg AEE. AEE (purity 99.5%) was provided by the Lanzhou Institute of Husbandry and Pharmaceutical Sciences of CAAS, and its concentration was based on our team’s previous studies (38). From the age of 14 days, broilers in the LPS and LPS + AEE groups received intraperitoneal injections of 0.5 mg/kg B.W LPS in saline once a day for 7 days; broilers in the saline and saline + AEE groups received peritoneal injection of the same dose of saline only once a day. Continuous injection for 7 days. This immune stress model induction method is based on our team’s previous experiments and improved (37). The experiment was conducted in accordance with the NRC guidelines (Table 1) for feeding a basal diet and in accordance with animal ethics guidelines and protocols approved by the Animal Care and Use Committee of Henan University of Science and Technology.

**Table 1:** Composition and nutrient levels of the experimental basal diet.

Ingredients, %	Content
Corn	60.1
Soybean meal	33.07
Soybean oil	3.6
Limestone-calcium carbonate	1.1
Calcium hydrogen phosphate	1
DL-methionine (98%)	0.2
L-lysine HCL (78%)	0.2
Sodium chloride	0.3
Vitamin Premix^a^	0.03
Mineral Premix^b^	0.2
Choline chloride (50%)	0.15
Ethoxyquin (33%)	0.05
Total	100
**Calculated nutrient levels** ^c^	
Metabolizable energy, kcal/kg	2,990
Crude protein	20.5
Calcium	0.99
Available phosphorus	0.44
Lysine	0.1
Methionine	0.47

a Vitamin premix provided the following per kg of diet: vitamin A (retinylacetate), 12,500 IU; vitamin D_3_ (cholecalciferol), 2,500 IU; vitamin E (DL-a-tocopherol acetate), 18.75 mg; vitamin K_3_ (menadione sodium bisulfate), 2.65 mg; vitamin B_1_, 2 mg; vitamin B_2_, 6 mg; vitamin B_6_, 6 mg; vitamin B_12_ (cyanocobalamin), 0.025 mg; biotin, 0.0325 mg; folic acid, 1.25 mg; pantothenic acid, 1.25 mg; nicotinic acid, 50 mg.

b Mineral premix provided per kilogram of complete diet: Cu (as copper sulfate), 8 mg; Zn (as zinc sulfate), 75 mg; Fe (as ferrous sulfate), 80 mg; Mn (as manganese sulfate), 100 mg; Se (as sodium selenite), 0.15 mg; I (as potassium iodide), 0.35 mg.

c Calculated value based on the analyzed data of experimental diets.

### 2.2 Sample collection

According to our team’s previous experiments (2), at 2 h, 4 h, 24 h after the initial injection (14d-2 h, 14d-4 h, 15d) and at 24 h after the 16d, 18d, 20d injections (17d, 19d, 21d), one chicken was selected from each cage for euthanasia and samples of ileal tissue and contents were collected. Part of the tissue samples was fixed in 4% paraformaldehyde, and the remaining tissue samples and contents were frozen in liquid nitrogen and transferred to −80°C for preservation.

### 2.3 Examination of intestinal morphology

The fixed ileum tissues were trimmed, dehydrated, embedded in paraffin, sectioned, stained with hematoxylin and eosin (H&E), microscopically examined and imaged using CaseViewer2.4 software to record the morphology and structure of the ileum. Ten relatively complete villi were selected from each ileum tissue section, and the villus height and crypt depth were measured. The mean value was determined and the ratio of villus height to crypt depth (V/C) was calculated.

### 2.4 Gene expression analysis using quantitative real-time PCR

TRIzol reagent (Thermo Fisher Scientific, Ottawa, Canada) was used to extract total RNA from ileum tissue, and gel electrophoresis on 1.0% agarose and Nano-drop2000 (Thermo Scientific, Ottawa, Canada) absorbance measurements were used to determine RNA concentration and purity. RNA was converted to cDNA with an Evo M-MLV mix kit (Accurate Biology, AG11728, China). NCBI/Primer-BLAST was used to design target gene-specific primers (Table 2), which were used for quantitative real-time. The SYBR Green premixed ProTaq-HS qPCR kit (Accurate Biology, AG11701, China) was used to prepare the reaction mixes and qRT-PCR was performed on a CFX-Connect real-time PCR system (Bio-Rad Laboratories, Hercules, CA). The specific operation is according to the instruction manual. The 2^−ΔΔCt^ method was used to analyze the relative gene expression levels.

**Table 2:** Primers for RT-qPCR analysis.

Gene^a^	Primer sequence (5′ to 3′)^b^	GenBank number
*occludin*	F: ACGGCAGCACCTACCTCAA R: GGGCGAAGAAGCAGATGAG	XM_025144247.2
*ZO-1*	F: CTTCAGGTGTTTCTCTTCCTCCTC R: CTGTGGTTTCA TGGCTGGATC	XM_021098886.1
*claudin-1*	F: CATACTCCTGGGTCTGGTTGGT R: GACAGCCA TCCGCA TCTTCT	NM_001013611.2
*claudin-2*	F: CCTACATTGGTTCAAGCATCGTGA R: GATGTCGGGAGGCAGGTTGA	NM_001277622.1
*TNF-α*	F: GAGCGTTGACTTGGCTGTC R: AAGCAACAACCAGCTA TGCAC	NM_214022.1
*IL-1β*	F: ACTGGGCA TCAAGGGCTA R: GGTAGAAGA TGAAGCGGGTC	NM_214005.1
*IL-6*	F: GCTGCGCTTCTACACAGA R: TCCCGTTCTCA TCCA TCTTCTC	NM_204628.1
*IL-10*	F: AGAAATCCCTCCTCGCCAAT R: AAATAGCGAACGGCCCTCA	NM_001004414.2
*COX-2*	F: CCGAATCGCAGCTGAATTCA R: GAAAGGCCATGTTCCAGCAT	NM_001277664.2
*mPGES-1*	F: AGGCTCAGGAAGAAGGCATT R: CACAGCTCCAAGGAAGAGGA	NM_001194983.1
*GAPDH*	F: TGCTGCCCAGAACATCATCC R: ACGGCAGGTCAGGTCAACAA	NM_204305

a *TNF-α*, tumor necrosis factor-α; *IL-1β*, interleukin-1β; *IL-6*, interleukin-6; *IL-10*, interleukin-10; *COX-2*, cyclooxygenase-2; *mPGES-1*, microsomal prostaglandin E synthase-1; *GAPDH*, glyceraldehyde-3-phosphate dehydrogenase.

b F, forward primer; R, reverse primer.

### 2.5 Analysis of the ileum microbiota

Total microbial genomic DNA was extracted from ileum contents samples using the E.Z.N.A.^®^ soil DNA kit (Omega Bio-tek, Norcross, GA, United States) according to manufacturer’s instructions. The quality and concentration of DNA were determined by 1.0% agarose gel electrophoresis and absorbance measurements with a Nano-drop2000 spectrometer (Thermo Scientific, Ottawa, Canada); DNA aliquots were kept at −80°C for later testing. The hypervariable region, V3-V4, of the bacterial 16S rRNA gene was amplified with the following primer pairs (39).

338F (5′-ACTCCTACGGGAGGCAGCAG-3′)

806R (5′-GGACTACHVGGGTWTCTAAT-3′)

with an ABI GeneAmp^®^ 9700 PCR thermocycler (ABI, CA, United States). The PCR product was electrophoresed on a 2% agarose gel, purified using the AxyPrep DNA gel extraction kit (Axygen Biosciences, Union City, CA, United States), and quantified using a Quantus^™^ fluorometer (Promega, United States). Purified amplicons were pooled in equimolar amounts and paired-end sequencing was done on an Illumina MiSeq PE300 platform (Illumina, San Diego, United States) according to the standard protocols by Majorbio Bio-Pharm Technology Co. Ltd. (Shanghai, China). The raw sequencing reads were deposited into the NCBI sequence read archive (SRA) database (Accession No: PRJNA1052936). Raw FASTQ files were quality-filtered with Fastp version 0.19.6 (40) and merged by FLASH version 1.2.7 (41). The optimized sequences were clustered into operational taxonomic units (OTUs) using UPARSE 7.1 (42) with 97% sequence similarity level, and the most abundant sequence for each OTU was selected as representative. To minimize the effects of sequencing depth on alpha and beta diversity calculations, the number of 16S rRNA gene sequences from each sample were rarefied to 20,000, which still yielded an average Good’s coverage of 99.09%. The taxonomy of each representative OTU sequence was determined using the RDP classifier version 2.2 (43) against the 16S rRNA gene database (Silva v138) using a confidence threshold of 0.7. The community composition of each sample was calculated at different species classification levels. Bioinformatic analysis of the ileum microbiota was carried out using the Majorbio cloud platform.^1^

### 2.6 Statistical analysis

The experimental results were analyzed by one-way ANOVA using SPSS software (ver. 20.0 for Windows, SPSS Inc., Chicago, IL, United States) followed by Duncan’s multiple comparison tests. The data are expressed as mean ± standard error, and *p* < 0.05 indicates a significant difference.

## 3 Results

### 3.1 Effects of AEE on ileal morphology in immune-stressed broilers

**Figure 1:**
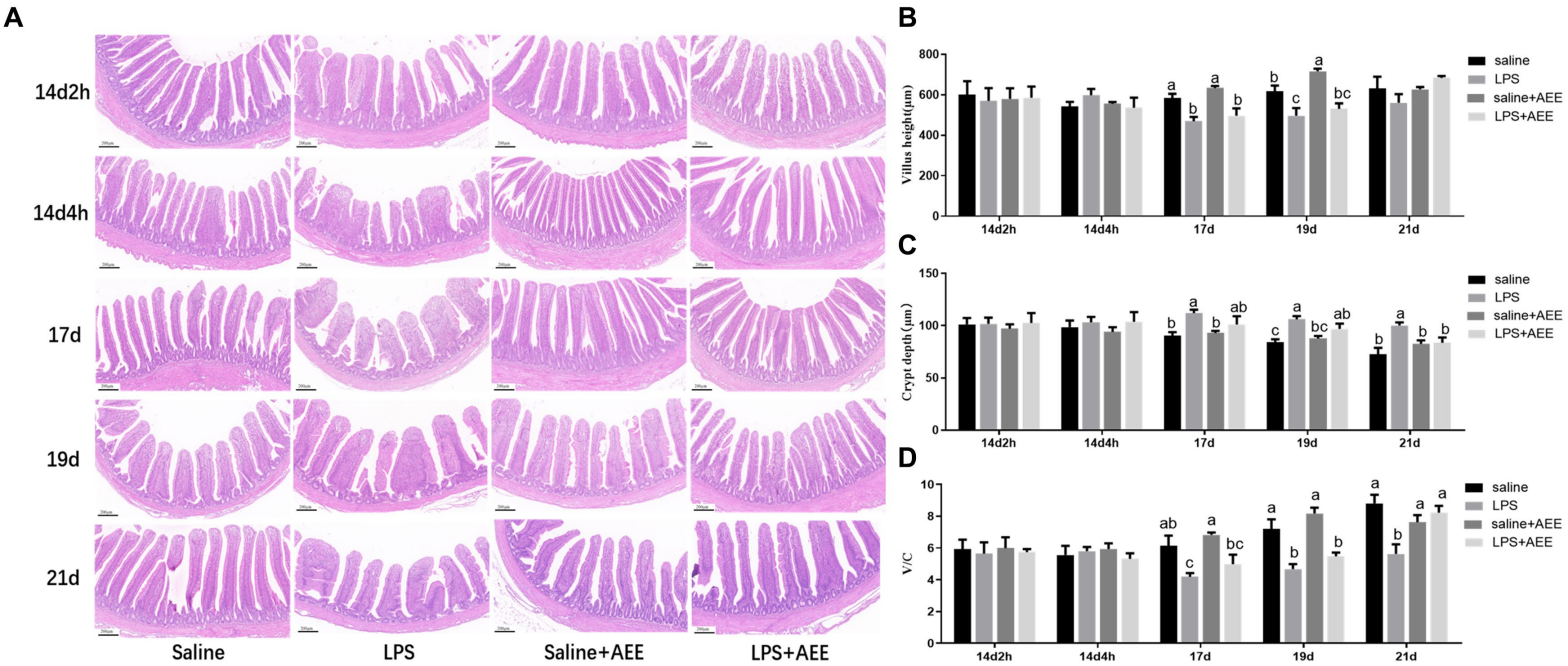
Effects of AEE on ileal morphology in immune-stressed broilers. **(A)** Morphological structure of ileal tissue. **(B)** Villus height. **(C)** Crypt depth. **(D)** V/C, ratio of villus height to crypt depth. Each vertical bar represents the mean ± SEM (*n* = 6). Bars with different letters differed significantly (*p <* 0.05). Scale bar, 200 μm.

The results of H&E staining (Figure 1A) showed that compared with saline group, the villi in the ileum from the LPS group were arranged sparsely, but feeding AEE alleviated the damage to the ileum morphology caused by immune stress. By measuring the villus height and crypt depth in the ileum, it was found that there was no significant difference in villus height between the groups at 14d-2 h, 14d-4 h, and 21d. At 17d and 19d, the villus height in the LPS group was significantly lower (*p <* 0.05) than that in the saline group (Figure 1B). From 17 to 21 days of age, the crypt depth in the LPS group was significantly greater (*p <* 0.05) than control, and it was reduced in the LPS + AEE group compared with the LPS group, while there was no significant difference in crypt depth between the saline+AEE group and the saline group (Figure 1C). The V/C calculation of the LPS group was lower (*p <* 0.05) than that of the saline group from 17 to 21 days of age, and V/C was increased by the addition of AEE to the diet (Figure 1D).

### 3.2 Effects of AEE on relative mRNA expression of tight junction genes in the ileum of immune-stressed broilers

**Figure 2:**
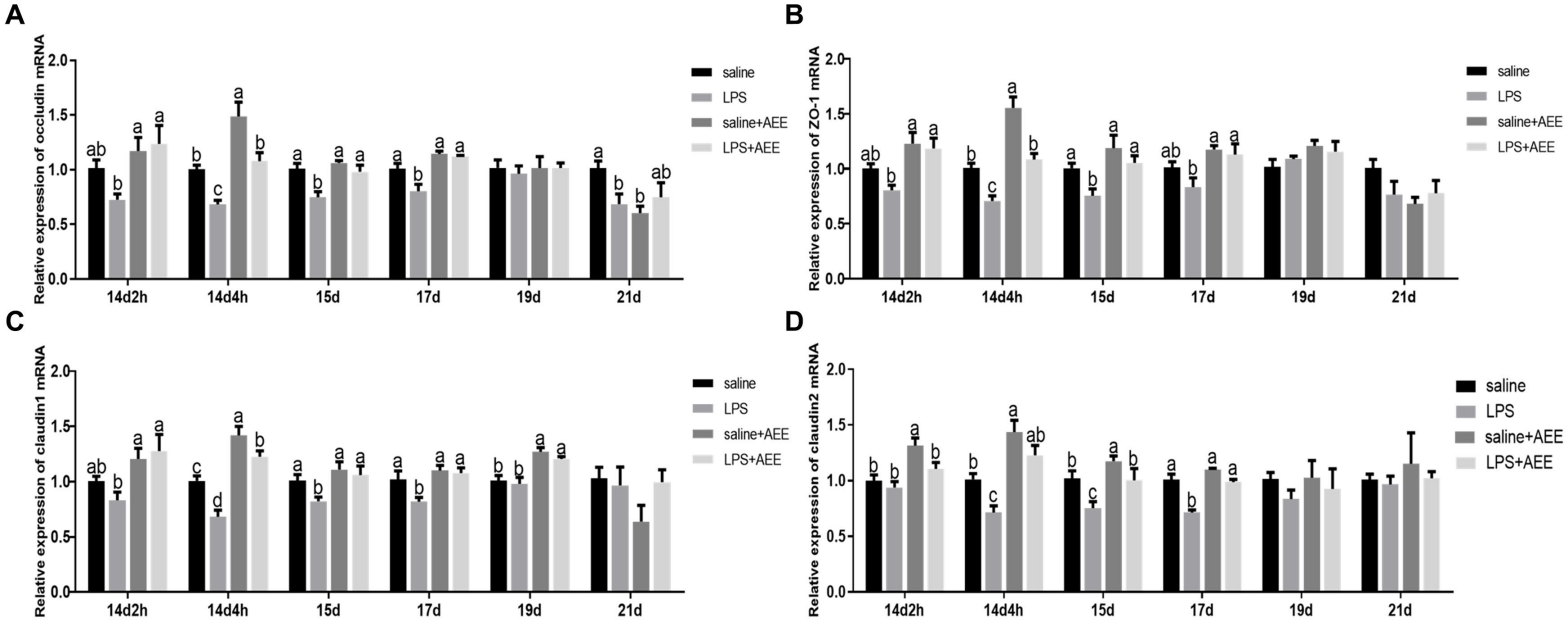
Effects of AEE on relative expression of tight junction protein mRNA in the ileum of immune-stressed broilers. Relative expression of *occludin* **(A)**, *ZO-1* **(B)**, *claudin-1* **(C)**, and *claudin-2* **(D)** mRNA in ileum. The gene for GAPDH was used as a reference for normalization. Each vertical bar represents the mean ± SEM (*n* = 6). Bars with different letters differed significantly (*p <* 0.05).

At 14d-2 h, there was no significant difference in the expression of the tight junction genes *occludin* (Figure 2A), zonula occludens-1 (*ZO-1*) (Figure 2B), *claudin-1* (Figure 2C) and *claudin-2* (Figure 2D) between the LPS group and the saline+AEE group compared to the saline group. From 14d-4 h to 17d, the expression of the tight junction genes in the LPS group was significantly lower (*p <* 0.05) than that in the saline group, and the addition of AEE to the feed significantly upregulated (*p <* 0.05) the expression of these genes. At 19d, the expression of *claudin-1* in the AEE added groups was significantly higher (*p <* 0.05) than that in the non-addition groups (Figure 2C).

### 3.3 Effects of AEE on relative mRNA expression of inflammatory cytokines in the ileum of immune-stressed broilers

**Figure 3:**
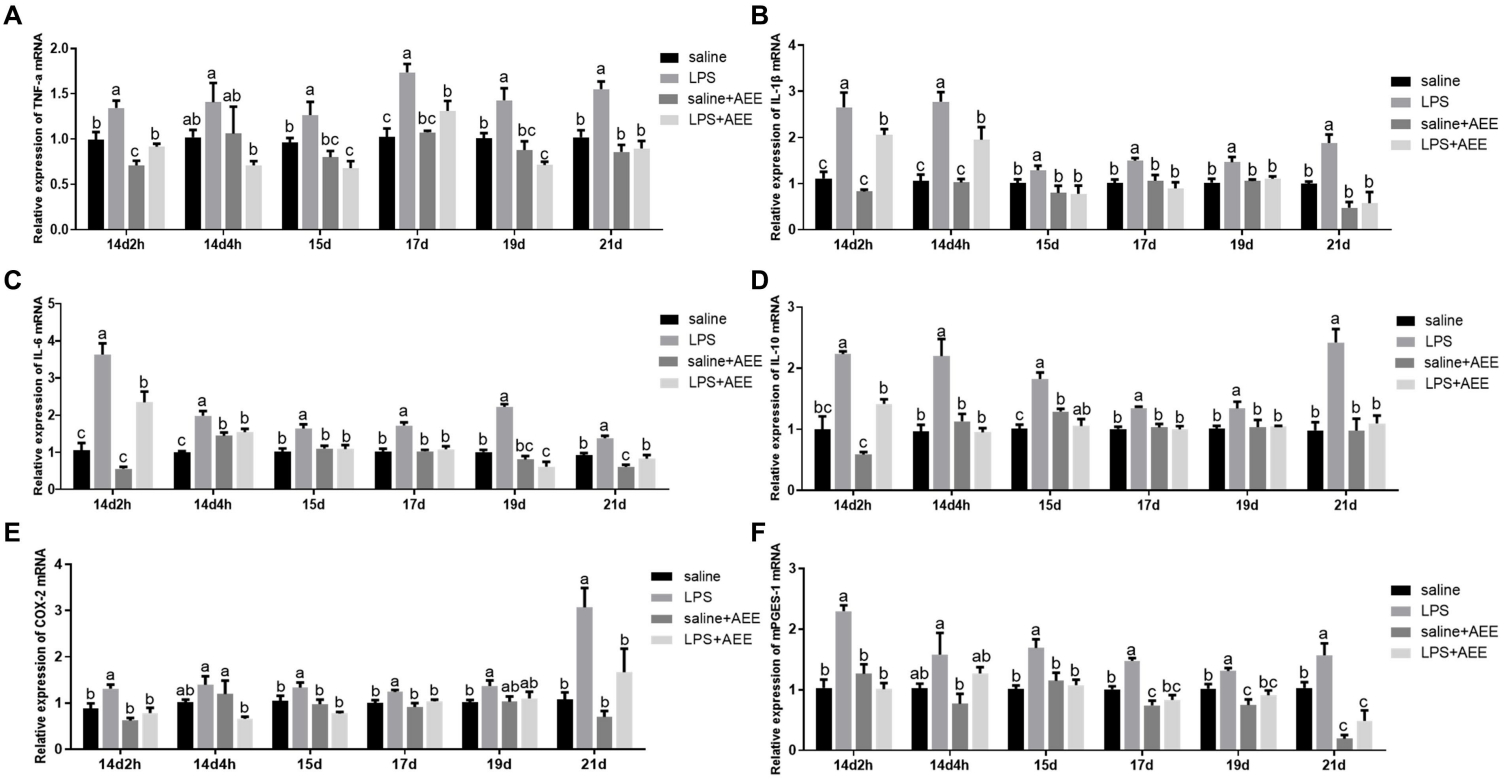
Effects of AEE on relative mRNA expression of inflammatory cytokines in the ileum of immune-stressed broilers. Relative mRNA expression of *TNF-α* **(A)**, *IL-1β* **(B)**, *IL-6* **(C)**, *IL-10* **(D)**, *COX-2* **(E)**, and *mPGES-1* **(F)** in ileum. The gene for GAPDH was used as a reference for normalization. Each vertical bar represents the mean ± SEM (*n* = 6). Bars with different letters differed significantly (*p <* 0.05).

Compared with saline group, LPS-induced immune stress significantly increased (*p <* 0.05) the relative mRNA expression of ileal inflammatory factors tumor necrosis factor-α (*TNF-α*) (Figure 3A), interleukin-1β (*IL-1β*) (Figure 3B), interleukin-6 (*IL-6*) (Figure 3C) and interleukin-10 (*IL-10*) (Figure 3D), *COX-2* (Figure 3E) and *mPGES-1* (Figure 3F) in the ileum of broilers. *TNF-α* in the LPS group showed high expression throughout the experiment, while the relative expression of *IL-1β*, *IL-6*, *IL-10*, and *mPGES-1* on 14d were higher than that at the later stages, and the relative expression of *COX-2* on 21d were higher than that at other time points. The expression of *TNF-α*, *IL-1β*, *IL-6*, *IL-10*, *COX-2*, and *mPGES-1* in the LPS + AEE group was significantly lower (*p <* 0.05) than that in LPS group, and the differences were obvious at each time point.

### 3.4 Effects of AEE on ileal microbiota composition and diversity in immune-stressed broilers

**Figure 4:**
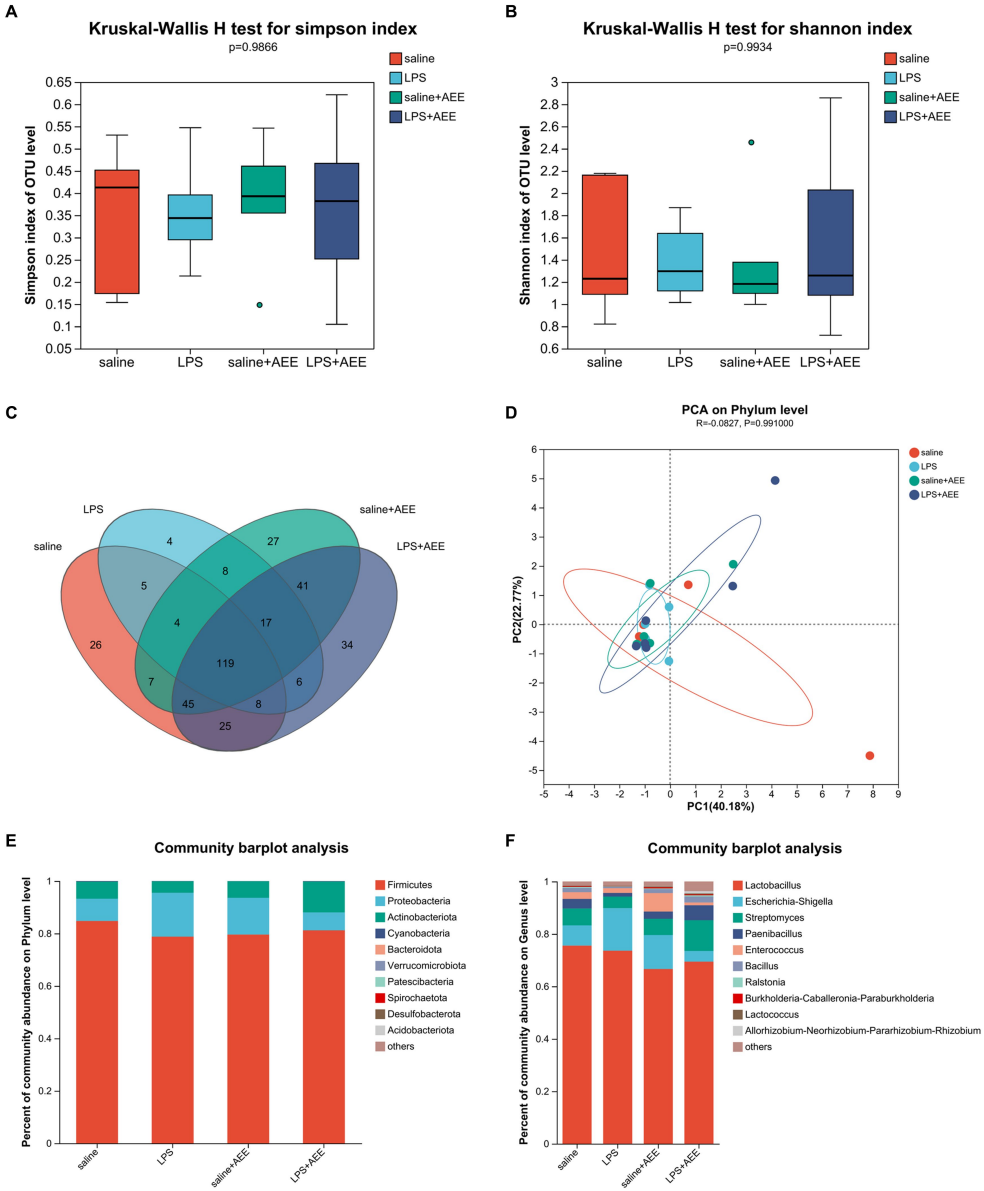
Effects of AEE on ileal microbiota composition and diversity in immune-stressed broilers. Alpha-diversity indicated by observed Simpson **(A)** and Shannon **(B)** index. Venn diagram showing overlap of compositions of bacterial OTUs in different groups **(C)**. Beta-diversity indicated by principal component analysis (PCA) on the phylum level **(D)**. Average relative abundances of dominant bacterial phylum **(E)** and genus level **(F)** in ileum under different treatments.

Alpha diversity analysis showed that there were no significant differences in the Simpson and Shannon indices of ileal microbiota among the four groups at 21d (Figures 4A,B). Venn diagram analysis (Figure 4C) of the composition of OTUs in the ileal microbiota at 21d showed that the number of OTUs shared by the four groups was 119. The number of unique OTUs in the saline, LPS, saline+AEE and LPS + AEE groups was 26, 4, 27, and 34, respectively. Principal Component Analysis (PCA) (Figure 4D) showed that the distribution of community composition among the samples of the four treatment groups was relatively concentrated. Community composition analysis showed that the predominant phyla in the ileum of the four groups (Figure 4E) were Firmicutes (84.77, 78.79, 79.59, 81.22%), Proteobacteria (8.51, 16.72, 14.04, 6.79%), and Actinobacteriota (6.50, 4.40, 6.28, 11.81%). Compared with the saline and LPS + AEE groups, the proportion of Firmicutes and Actinobacteriota in the LPS group was lower, while the proportion of Proteobacteria was higher. At the genus level (Figure 4F), the dominant genera in the ileum of the four groups were *Lactobacillus* (75.55, 73.61, 66.63, 69.43%), *Escherichia-Shigella* (7.71, 16.24, 12.94, 4.08%), *Streptomyces* (6.43, 4.39, 6.20, 11.71%), *Paenibacillus* (3.66, 1.41, 2.76, 5.70%), *Enterococcus* (2.57, 1.79, 7.06, 1.04%), *Bacillus* (1.62, 0.52, 1.46, 2.29%), *Ralstonia* (0.41, 0.19, 0.33, 0.50%), *Burkholderia-Caballeronia-Paraburkholderia* (0.28, 0.11, 0.44, 0.31%), and *Lactococcus* (0.23, 0.16, 0.31, 0.35%). Compared with the saline group, the proportion of *Lactobacillus*, *Streptomyces*, *Paenibacillus*, *Enterococcus* and *Bacillus* in the LPS group was lower. The proportion of *Escherichia-Shigella* in the LPS group was higher than that in the saline group and the LPS + AEE group. The proportion of *Streptomyces*, *Paenibacillus* and *Bacillus* in the LPS + AEE group was higher than in the LPS group.

### 3.5 Line discriminant analysis effect size analysis of ileal microbiota

**Figure 5:**
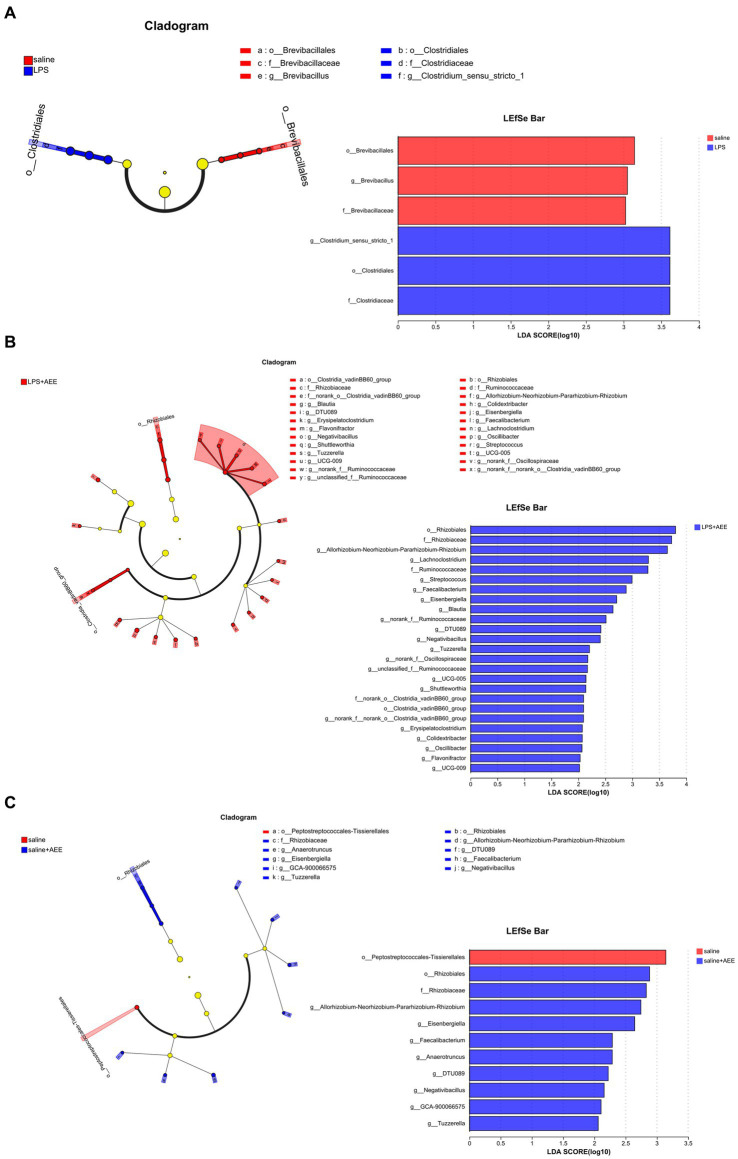
Cladograms and bar plots obtained by LEfSe analysis showed differences in microbiota between the treatment groups. LEfSe analysis of the saline and LPS groups **(A)**. LEfSe analysis of the LPS and LPS + AEE groups **(B)**. LEfSe analysis of the saline and saline + AEE groups **(C)**. The different color nodes in cladogram represent the microbial groups that are significantly enriched in the corresponding group and have a significant effect on the difference between the groups. Light yellow nodes represent microbial groups that do not differ significantly between groups, or have no significant effect on differences between groups. The LDA scores represent the effect size of each abundant species. The bar plots show the microbiota with LDA score > 2, which is a biomarker of statistical difference (LDA score threshold = 2).

By LEfSe analysis of the saline and LPS groups, the saline group was enriched in Brevibacillales, Brevibacillus and Brevibacillaceae, while the LPS group was enriched in *g_Clostridium_sensu_stricto_1*, o_Clostridiales, f_Clostridiaceae (Figure 5A). Compared with the LPS group, the LPS + AEE group was enriched in Rhizobiales, Rhizobiaceae, Allorhizobium-Neorhizobium-Pararhizobium-Rhizobium, *Lachnoclostridium*, Ruminococcaceae, Streptococcus, Faecalibacterium, Eisenbergiella, Blautia, DTU089, Negativibacillus, Tuzzerella, Oscillospiraceae, UCG-005, Shuttleworthia, Clostridia_vadinBB60_group, Erysipelatoclostridium, Colidextribacter, Oscillibacter, Flavonifractor and UCG-009 (Figure 5B). Compared with the saline group, the saline+AEE group was enriched in Rhizobiales, Rhizobiaceae, Allorhizobium-Neorhizobium-Pararhizobium-Rhizobium, Eisenbergiella, Faecalibacterium, Anaerotruncus, DTU089, Negativibacillus, GCA-900066575 and Tuzzerella (Figure 5C).

## 4 Discussion

The gut is not only a digestive organ, but also has a powerful immune function in the body (44). The normal structure of the intestinal mucosa is designed for digestion (45) and nutrient absorption, but also has immunoregulatory activity. Intestinal villus height, crypt depth and their ratio (V/C) are important indicators of intestinal health. The restoration of normal intestinal morphology and structure can help relieve stress and improve intestinal barrier function (46). The higher the villus height, the larger the surface area available for nutrient absorption (47). A shallower crypt depth indicates more mature cells (48) with superior ability to digest and absorb beneficial compounds (49). The V/C ratio reflects the comprehensive intestinal nutrient absorption capacity and the degree of development (50). Broilers under intensive production conditions are easily infected by pathogenic microorganisms, which often induce immune stress in the chickens (51), causing damage to the intestinal mucosa (52), intestinal villi atrophy (53), increased crypt depth and intestinal permeability (54). Nie et al. (55) found that LPS-induced immune stress increased intestinal permeability, damaged mucosal structure, increased crypt depth, and decreased villus height and V/C in chickens. Similarly, in this study, peritoneal injection of broilers with LPS seriously damaged ileum morphology, and resulted in a significant decrease in villus height on 17d and 19d, a significant increase in crypt depth from 17d to 21d, and a significant decrease in V/C. However, dietary supplementation with AEE significantly reduced the morphological damage and the villus crypt depth, and increased V/C in the later stages of the experiment. This suggests that AEE has a potential protective effect on intestinal barrier function and can promote intestinal nutrient absorption by improving ileum villi morphology.

Tight junctions are the main connective structures between intestinal mucosal epithelial cells (56), and they play a critical role in maintaining the integrity of the intestinal mucosal structure and a strong intestinal barrier (57). The tight junctions between intestinal epithelial cells are formed by a number of specific proteins such as *occludin*, *claudins*, *ZOs* and the junctional adhesion molecule (58). Occludin and *claudin-1* are mainly employed in the construction and maintenance of tight junction structures (59), while *claudin-2* forms cell bypass pores and participates in water transport and ion transfer (60). *ZO-1* is an important scaffold protein, which regulates adhesion junctions and signal transduction between cells by interacting with other tight junction proteins (61). When broilers are subjected to immune stress, the connections between the ileal epithelial cells weaken, allowing more inflammatory pathogen molecules and disease-causing bacteria to pass through and disrupt intestinal immune function (62). According to a report (63), LPS stimulation significantly decreased the expression of *occludin*, *ZO-1* and *claudin-1* in the ileum of broilers, and induced intestinal barrier dysfunction. In this study, LPS-induced immune stress significantly reduced the relative expression of the tight junction genes *occludin*, *ZO-1*, *claudin-1*, and *claudin-2* in the ileum at 14d-4 h, 15d, and 17d, which is evidence of impaired intestinal barrier function. Dietary AEE supplementation significantly increased the expression of these tightly linked genes in the ileum of immune-stressed broilers, especially in the early stages of the experiment. Taken together, these results suggest that AEE has a positive regulatory effect on intestinal tight junction gene expression, thereby improving intestinal barrier function.

Intestinal barrier damage is often associated with an inflammatory response (64), and signals from the inflammatory cytokines are key to the regulation of the inflammatory response (65). *TNF-α* and *IL-1β* are important pro-inflammatory cytokines in the intestine (66), and *IL-6* has both pro-inflammatory and anti-inflammatory regulatory properties, depending on the environment in which it is produced and released (67). *IL-10* is a critical anti-inflammatory cytokine with immunomodulatory functions (68), which can inhibit the expression of *TNF-α*, *IL-6*, and *IL-1β* (69). When pro-inflammatory cytokines are expressed in large quantities, the body can lower the inflammatory response by up-regulating the expression of anti-inflammatory cytokines (70), which jointly participate in maintaining the immune balance and barrier function of the intestine. The results of this study showed that intraperitoneal injection of LPS significantly enhanced the mRNA expression of the ileal inflammatory factors *TNF-α*, *IL-1β*, *IL-6*, and *IL-10* throughout the trial. However, increased levels of IL-10 mRNA expression are not always beneficial. In some cases, the high expression of IL-10 may inhibit the effective clearance of pathogens by the immune system, leading to the persistence or aggravation of infection. In addition, in some autoimmune diseases, the regulatory function of IL-10 may be impaired, making inflammation still unable to be effectively controlled even when the IL-10 mRNA expression level is increased. Therefore, the results of this study fully confirmed the LPS-mediated intestinal inflammatory response in broilers. Consistent with our results, Liu et al. (71) showed that LPS treatment led to increased mRNA expression of *TNF-α*, *IL-1β*, and *IL-6* in the ileum of broilers. LPS can induce systemic inflammatory response in animals by regulating I-κB kinase/NF-κB, Toll-like receptors and downstream cytokine genes signaling pathways (72). In this study, the expression of ileal inflammatory factor genes in the LPS + AEE group was significantly down-regulated compared with the LPS group, suggesting that AEE had a significant alleviating effect on ileal inflammation in broilers exposed to immune stress. Similar studies have confirmed that AEE shows good anti-inflammatory properties both *in vivo* and *in vitro* (20). It can inhibit the formation of LPS-induced inflammatory mediators, and significantly reduce the expression and secretion of inflammatory cytokines such as *IL-1β*, *TNF-α*, and *IL-6*, thus alleviating the inflammatory response (18). Thus, our study strongly supports the anti-inflammatory potential of AEE in broilers and provides a theoretical basis for the role of AEE in resisting immune stress.

It is well known that stress can activate the Hypothalamic–Pituitary–Adrenal (HPA) axis, and our previous studies have found that the HPA axis can be activated by up-regulation of the expression of the *COX-2*/*mPGES-1*/*PGE_2_* signaling pathway in LPS-induced immune stress models (73). In this study, we obtained the same results: the expression of *COX-2* and its downstream enzyme *mPGES-1* in the ileum of broilers in the LPS group was significantly higher than that in saline group. *COX-2* is only expressed at low levels in most tissues and organs in a healthy body. It is induced by cytokines at the site of inflammation and injury, causing the synthesis and accumulation of prostaglandins at the damaged site, promoting an inflammatory response leading to tissue damage (74), which is consistent with the above elevated expression of ileum inflammatory factor genes in broilers treated with LPS. These results suggest that ileal inflammation induced by peritoneal injection of LPS may also be related to the increased mRNA levels of *COX-2* and *mPGES-1* in broilers. The *COX-2*-related signaling pathway can also regulate the expression of intestinal tight junction proteins (75). This would affect the integrity of the intestinal epithelial barrier, suggesting that the decrease in tight junction gene expression caused by LPS stimulation may involve the activation of the *COX-2*-related signaling pathway. Previous studies have confirmed that AEE can regulate the pathways associated with arachidonic acid metabolism and reduce the expression of *COX-2* and other genes (20). Similarly, the data of this study showed that the addition of AEE significantly decreased the mRNA expression of *COX-2* and *mPGES-1* in the ileum of broilers under LPS-induced immune stress. Thus, we further confirmed the protective effect of AEE on intestinal health in immune-stressed broilers by alleviating ileal inflammation and improving barrier function through a mechanism that may involve *COX-2*-related signal transduction.

The current study also revealed that the composition of gut microbes can significantly affect the health of poultry (76). The intestinal microbiota plays an important role in inhibiting pathogen infection and regulating digestion and nutrient absorption (77). The beneficial microbes help to maintain homeostasis by protecting the intestinal barrier (78) and regulating the nervous, endocrine and immune systems, which are indispensable parts of the body (79, 80). Under normal conditions, the intestinal microbiota of broilers is in a relatively stable equilibrium, with the beneficial gut bacteria colonizing the intestinal mucosa, thus preventing the attachment and growth of pathogenic bacteria and promoting the optimal regulation of the immune system and other physiological processes (81, 82). However, when the body is stressed, the harmful bacteria in the intestine multiply rapidly and produce a large amount of bacterial endotoxin, which causes an imbalance in the normal microbial community in the gut (83), changes the normal physiological and biochemical environment of the intestine, negatively affects normal intestinal function, and can lead to host disease (84). In this study, immune stress reduced the relative abundance of beneficial bacteria, such as Firmicutes, and increased the relative abundance of harmful bacteria, such as Proteobacteria, in the ileum of broilers. Firmicutes are a common type of dominant bacteria in the intestines of broilers (85). They participate in the host’s material and energy metabolism processes (86), produce butyrate that promotes the development of intestinal epithelial cells and has anti-inflammatory effects (87), and they are capable of digesting dietary fiber and other food components, and interacting with the intestinal mucosa to protect health (88). There are many pathogenic microorganisms in the phylum Proteobacteria, such as *Helicobacter pylori*, *Escherichia* and *Salmonella* (89), and an increase in Proteobacteria is a sign of gut bacterial imbalance (90). On the genus level, the proportion of beneficial bacteria such as *Lactobacillus* in the ileum of the LPS group was lower than that in the saline group, while the proportion of *Escherichia-Shigella* was higher. *Lactobacillus* is a common probiotic in the phylum Bacteroidota (91), which can ferment in the intestine to produce lactic acid, reduce intestinal pH to inhibit the growth of harmful bacteria, and effectively maintain the acid–base balance of the intestine (92). It can also help to enhance immune function and provide nutritional support to promote intestinal health (93). *Escherichia-Shigella* can cause gastrointestinal infections such as diarrhea and food poisoning (94). This further suggests that LPS-induced immune stress can lead to intestinal microecological imbalance. In this study, the addition of AEE increased the proportion of Firmicutes and Lactobacillus in the ileum of broilers stimulated by LPS, and reduced the proportion of Proteobacteria and *Escherichia-Shigella*, which proves that AEE can regulate the ileum microbiota of broilers under immune stress, alleviate the microbial imbalance caused by LPS stimulation, and improve intestinal health. This is consistent with the findings of Ma et al. (25) and Lu et al. (26). LEfSe analysis showed that *Brevibacillus* was dominant in the saline group compared with the LPS group, and we found that bacillus fermentation products could improve intestinal morphology and growth performance, increase short-chain fatty acid (SCFA) level, normalize gut microbial composition and maintain optimal intestinal health of broilers (95). Compared with the LPS group, the LPS + AEE group was rich in *Rhizobium*, *Lachnoclostridium*, Ruminococcaceae, *Faecalibacterium*, *Negativibacillus*, Oscillospiraceae, *Flavonifractor*, and others that have also been shown to play an important role in maintaining health (96–99). β-glucan extracted from *Rhizobium* can promote growth and immune regulation and can control obesity (100). *Lachnoclostridium* has important metabolic and immunomodulatory functions in the intestinal microbiota, and its abundance is positively correlated with the level of acetic acid in the intestine, which can effectively stabilize the intestinal environment through anti-inflammatory and immunosuppressive effects (101). Ruminococcaceae can break down plant cellulose and other complex carbohydrates and produces short-chain fatty acids, such as butyric acid and acetic acid, which are essential for maintaining gut health (102). *Faecalibacterium* plays an important role in promoting intestinal barrier function and inhibiting inflammation (103). Taken as a whole, the results of these experiments demonstrate that the addition of AEE can improve the intestinal bacterial composition of broilers, thereby contributing to the improvement of digestion, absorption and immune function.

## 5 Conclusion

Supplementation of broiler diets with 0.1 g/kg AEE protected intestinal health by improving intestinal villus morphology, enhancing tight junction gene expression and reducing the inflammatory response and immune stress of broilers caused by LPS stimulation, and the mechanism may be related to *COX-2* signal transduction and improved intestinal microbiota composition.

## Data availability statement

The data presented in the study are deposited in the https://www.ncbi.nlm.nih.gov/, accession number PRJNA1052936.

## Ethics statement

The animal study was approved by Animal Care and Use Committee of Henan University of Science and Technology. The study was conducted in accordance with the local legislation and institutional requirements.

## Author contributions

RZ: Data curation, Formal analysis, Investigation, Writing – original draft. DB: Investigation, Writing – original draft. WZ: Data curation, Formal analysis, Writing – review & editing. XH: Data curation, Formal analysis, Writing – review & editing. HZ: Data curation, Formal analysis, Writing – review & editing. JZ: Data curation, Formal analysis, Writing – review & editing. YZ: Writing – review & editing. KI: Writing – review & editing. BZ: Writing – review & editing. YY: Supervision, Writing – review & editing. JL: Supervision, Writing – review & editing. YM: Project administration, Writing – review & editing.

## Footnotes

^1^ https://cloud.majorbio.com
